# Supplementary material for: PARROT: Prediction of enzyme abundances using protein-constrained metabolic models
Source: PLoS Comput Biol. 2023 Oct 19;19(10):e1011549. doi: 10.1371/journal.pcbi.1011549 (PMC10617714; doi:10.1371/journal.pcbi.1011549)
Supplement: S1 Table — (DOCX) [file pcbi.1011549.s001.docx]

**S1 Table** – Experimental proteomics measurements used for yeast

| **Condition** | **Usage by PARROT** | **Reference** |
| --- | --- | --- |
| Lahtvee2017_REF | Reference | [1] |
| Lahtvee2017_EtOH20 | Alternative |  |
| Lahtvee2017_EtOH40 |  |  |
| Lahtvee2017_EtOH60 |  |  |
| Lahtvee2017_Osmo02 |  |  |
| Lahtvee2017_Osmo04 |  |  |
| Lahtvee2017_Osmo06 |  |  |
| Yu2020_Clim | Reference | [2] |
| Yu2020_CN30 | Alternative |  |
| Yu2020_CN50 |  |  |
| Yu2020_CN115 |  |  |
| Yu2021_std_010 | Reference | [3] |
| Yu2021_N30_005 | Alternative |  |
| Yu2021_N30_010 |  |  |
| Yu2021_N30_013 |  |  |
| Yu2021_N30_018 |  |  |
| Yu2021_N30_030 |  |  |
| Yu2021_N30_035 |  |  |
| Yu2021_Gln_glc1 | Reference |  |
| Yu2021_Gln_glc2 | Alternative |  |
| Yu2021_Gln_N30 |  |  |
| Yu2021_Phe_std | Reference |  |
| Yu2021_Phe_N30 | Alternative |  |
| Yu2021_Ile_std | Reference |  |
| Yu2021_Ile_N30 | Alternative |  |
|  |  |  |

**References**

1. Lahtvee PJ, Sánchez BJ, Smialowska A, Kasvandik S, Elsemman IE, Gatto F, et al. Absolute Quantification of Protein and mRNA Abundances Demonstrate Variability in Gene-Specific Translation Efficiency in Yeast. Cell Syst. 2017;4: 495-504.e5. doi:10.1016/j.cels.2017.03.003

2. Yu R, Campbell K, Pereira R, Björkeroth J, Qi Q, Vorontsov E, et al. Nitrogen limitation reveals large reserves in metabolic and translational capacities of yeast. Nat Commun. 2020;11: 1–12. doi:10.1038/s41467-020-15749-0

3. Yu R, Vorontsov E, Sihlbom C, Nielsen J. Quantifying absolute gene expression profiles reveals distinct regulation of central carbon metabolism genes in yeast. Elife. 2021;10. doi:10.7554/ELIFE.65722
