# Supplementary material for: PARROT: Prediction of enzyme abundances using protein-constrained metabolic models
Source: PLoS Comput Biol. 2023 Oct 19;19(10):e1011549. doi: 10.1371/journal.pcbi.1011549 (PMC10617714; doi:10.1371/journal.pcbi.1011549)
Supplement: S2 Table — (DOCX) [file pcbi.1011549.s002.docx]

**S2 Table** – Experimental proteomics measurements used for *Escherichia coli*

| **Condition** | **Usage by PARROT** | **Reference** |
| --- | --- | --- |
| GLYC_BATCH_mu=0.47_S | Reference | [1] |
| ACE_BATCH_mu=0.3_S | Alternative |  |
| GAM_BATCH_mu=0.46_S |  |  |
| GLC_BATCH_mu=0.58_S |  |  |
| MAN_BATCH_mu=0.47_S |  |  |
| PYR_BATCH_mu=0.4_S |  |  |
| XYL_BATCH_mu=0.55_S |  |  |
| GLC_CHEM_mu=0.12_S | Reference |  |
| GLC_CHEM_mu=0.20_S | Alternative |  |
| GLC_CHEM_mu=0.35_S |  |  |
| GLC_CHEM_mu=0.50_S |  |  |
| GLC_CHEM_mu=0.11_V | Reference | [2] |
| GLC_CHEM_mu=0.21_V | Alternative |  |
| GLC_CHEM_mu=0.31_V |  |  |
| GLC_CHEM_mu=0.40_V |  |  |
| GLC_CHEM_mu=0.49_V |  |  |
| GLC_CHEM_mu=0.21_P | Reference | [3] |
| GLC_CHEM_mu=0.22_P | Alternative |  |
| GLC_CHEM_mu=0.26_P |  |  |
| GLC_CHEM_mu=0.31_P |  |  |
| GLC_CHEM_mu=0.36_P |  |  |
| GLC_CHEM_mu=0.41_P |  |  |
| GLC_CHEM_mu=0.46_P |  |  |
| GLC_CHEM_mu=0.51_P |  |  |

**References**

1. Schmidt A, Kochanowski K, Vedelaar S, Ahrné E, Volkmer B, Callipo L, et al. The quantitative and condition-dependent *Escherichia coli* proteome. Nat Biotechnol. 2016;34: 104–110. doi:10.1038/nbt.3418

2. Valgepea K, Adamberg K, Seiman A, Vilu R. *Escherichia coli* achieves faster growth by increasing catalytic and translation rates of proteins. Mol Biosyst. 2013;9: 2344–2358. doi:10.1039/C3MB70119K

3. Peebo K, Valgepea K, Maser A, Nahku R, Adamberg K, Vilu R. Proteome reallocation in *Escherichia coli* with increasing specific growth rate. Mol Biosyst. 2015;11: 1184–1193. doi:10.1039/c4mb00721b
