# Supplementary figures and images for: PARROT: Prediction of enzyme abundances using protein-constrained metabolic models
Source: PLoS Comput Biol. 2023 Oct 19;19(10):e1011549. doi: 10.1371/journal.pcbi.1011549 (PMC10617714; doi:10.1371/journal.pcbi.1011549)

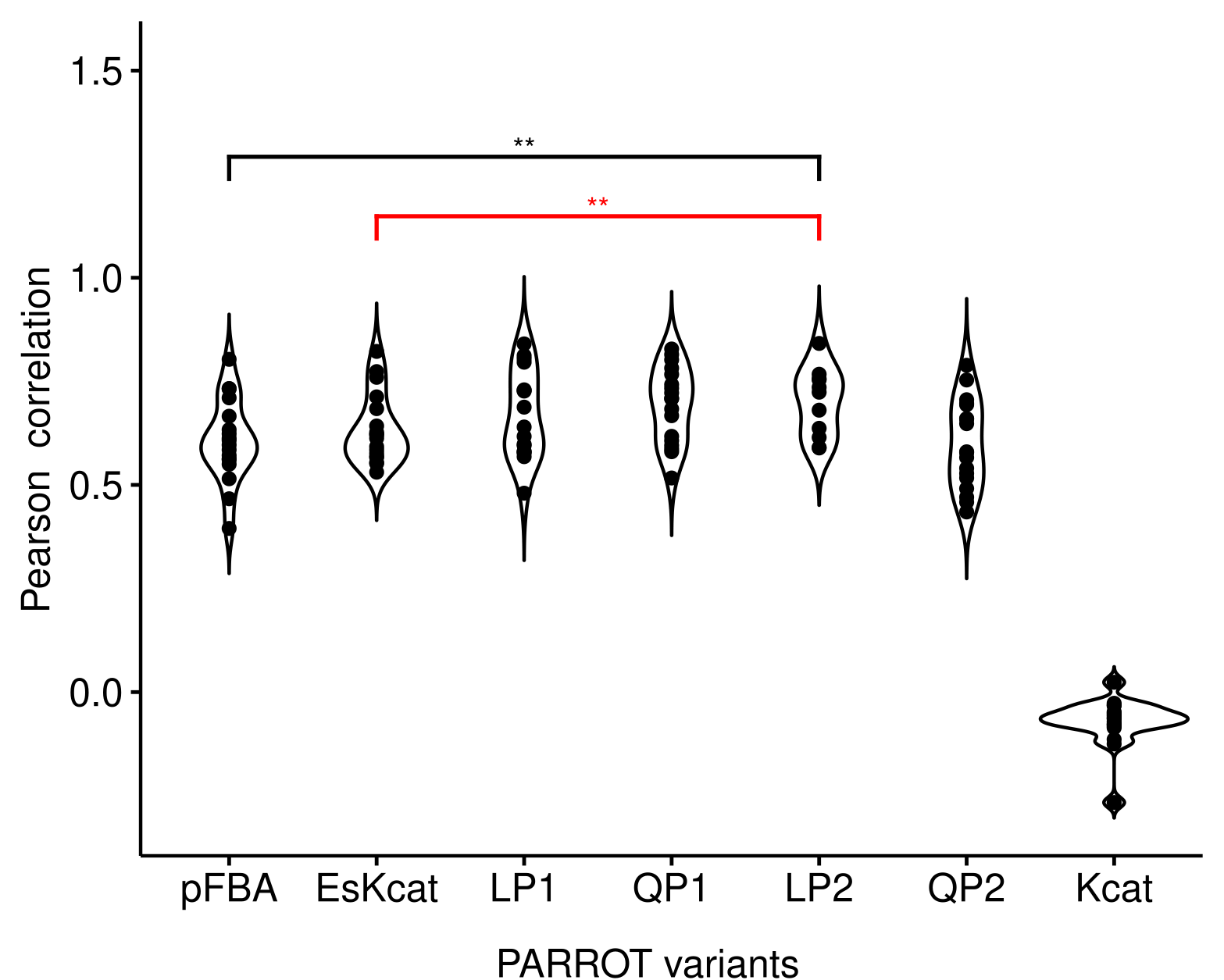

Supplement: S1 Fig — All values were log10-transformed prior to comparisons. A pairwise Wilcoxon rank sum assesses the statistical significance: ** p-value < 0.0009. Black significance bar indicates comparisons to pFBA. Red significance bar indicates comparisons to EsKcat. (TIFF) [file pcbi.1011549.s003.tiff]

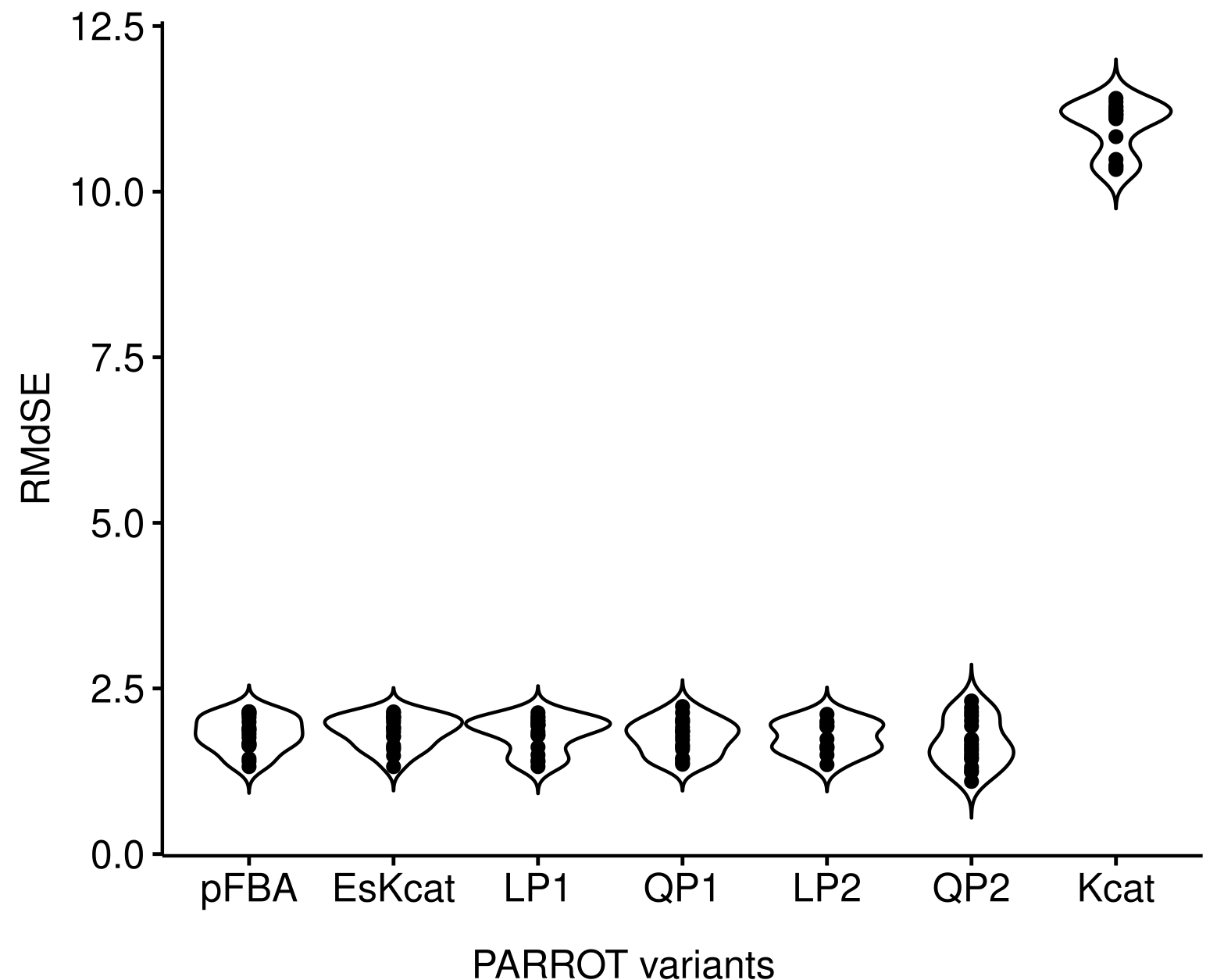

Supplement: S2 Fig — The minimization of the 2-norm of the experimental enzyme usage distribution in S. cerevisiae was used. All values were log10-transformed prior to comparisons. (TIFF) [file pcbi.1011549.s004.tiff]

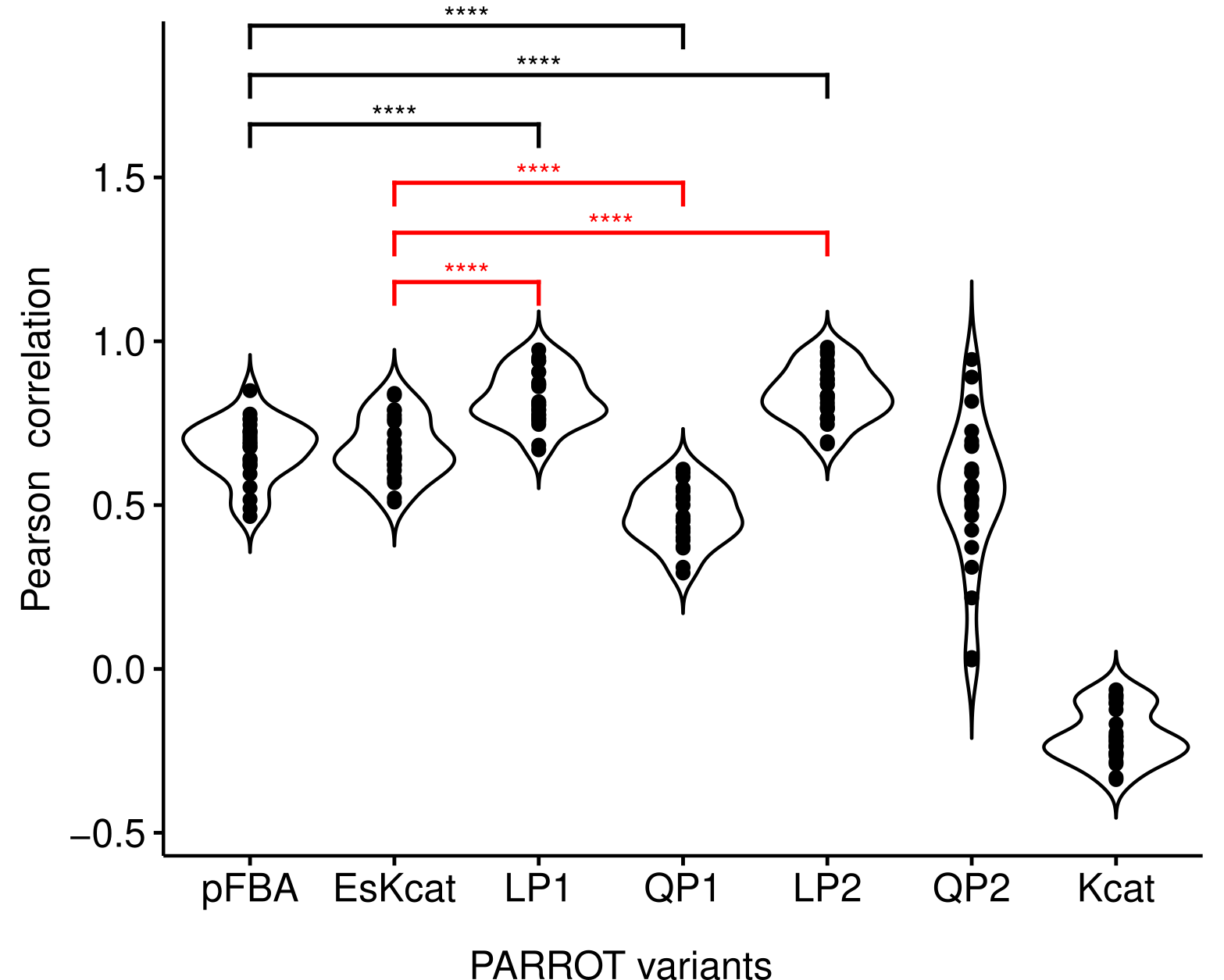

Supplement: S3 Fig — All values were log10-transformed prior to comparisons. A pairwise Wilcoxon rank sum assesses the statistical significance: **** p-value < 0.000005, * p-value < 0.03. Black significance bar indicates comparisons to pFBA. Red significance bar indicates comparisons to EsKcat. (TIFF) [file pcbi.1011549.s005.tiff]

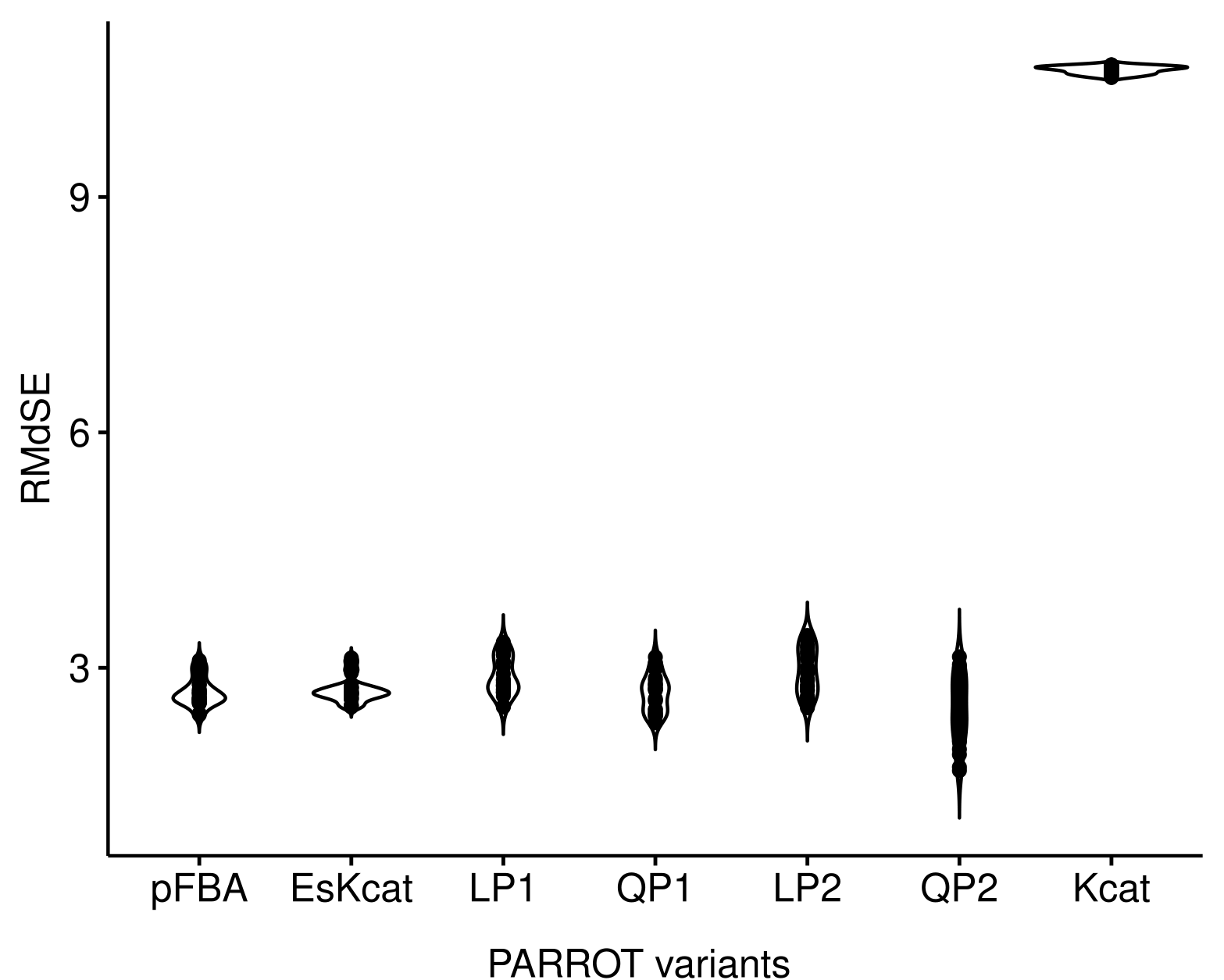

Supplement: S4 Fig — The minimization of the second norm of the experimental enzyme usage distribution in E. coli was used. All values were log10-transformed prior to comparisons. (TIFF) [file pcbi.1011549.s006.tiff]
