# Supplementary material for: PARROT: Prediction of enzyme abundances using protein-constrained metabolic models
Source: PLoS Comput Biol. 2023 Oct 19;19(10):e1011549. doi: 10.1371/journal.pcbi.1011549 (PMC10617714; doi:10.1371/journal.pcbi.1011549)
Supplement: Attachment — Submitted filename: parrot_comments.odt [file pcbi.1011549.s007.odt]

Review of PARROT


Major issue:
1. This submission claims to outperform the “competing algorithms” in predicting enzyme abundances, but does not actually compare to any software which in the original publication claims to predict enzyme abundances. It only compares its results to pFBA, which is a method that predicts fluxes. The authors extend this version to predict enzyme abundances, but this is so-far unpublished method which no-one claims can predict enzyme abundances. 
2. Lack of comparison to experimental data, and a biological analysis: do you see a particular metabolic adaptation that you would have missed by using pFBA alone? Where is the added value? Even a single example would suffice. 

Minor (but important) issues:
2. The Manhattan distance method, which the authors claim is the best performing one, is formalized directly as an LP (linear programming) problem. To my knowledge, absolute values cannot be directly put into a formulation of an LP problem. I would expect a further elaboration on this – how did you transform it to an actual LP problem?
3. The use of term “optimal growth condition” is vague. I would appreciate authors either defining what they mean by optimal. Comparing the usage of that term to the rest of their text, it would seem to me that optimal actually means “the control condition”.
One simple example: it can be that the control condition for the growth of E. coli is at 37 degrees Celsius, and the “suboptimal” condition is growth at 42 deg C (Schmidt data), but the cells actually grow faster at the “suboptimal” growth condition. 
4. Lack of information how you go from proteomics to enzyme abundances (is this what you use GECKO for?) It wasn’t completely clear to me. Protein and enzyme abundances are quite different things… I guess this is what you use “flexibilization” for, but I’d appreciate an explanation on what that actually means, since you never compare to proteomics directly, but always to this “flexibilized” baseline. Why this strange baseline?
5. Table with parameters (P_tots, growth rates, uptake rates). Some things are in the supplementary (growth rates), and P_tots are in your MATLAB code, but one table summarizing this could be of great value to future researchers. Also – could you tell me where in Schmidt data did you find total protein content – I remember this being quite hard to find and existent only for one condition – or am I wrong here?


On software:
There are no clear instructions on how to run the software on the author’s GitHub pages. I have downloaded and installed Cobra, Raven and Gecko, and am still unable to run their code.
The mistake I get is that there is no method called “FlexibilizeConstraints” in none of these packages (including PARROT). I have recursively “grepped” all of these directories searching for “def FlexibilizeFluxes” to find a file in which this has been defined, but unsuccessfully. 
I would (first) want to be able to run the code, and (second) to have a much more informative README file which informs me of an example run (a code which should work).

What I see as options:
- either compare your results to a published algorithm which claims to predict enzyme abundances
- or weaken the claim about “outperforming the competition” (which in your current formulation is not exactly true) and provide more information about how your predictions compare to experimental data.

To be clear – I do see something valuable and interesting in your approach, but I do not see that you have proven your claims about “outperforming the competition”. 

More detailed comments:
In your abstract you mention 2 times “enzyme allocation adjustment” and “enzyme reallocation”, but you fail to mention that this refers to a control condition. Only in the last sentence do you mention a “suboptimal” growth condition (a term I find very vague). This could leave the readers with a false impression that you predict per-condition proteome allocation, which is not exactly true.

Lines 27-28: Where do you show this inconsistency?

Lines 29-31: There are other approaches that claimed that and showed that it has predictive power. I do not see this as the big claim of your work. Just a few as examples:

Molenaar, Douwe, et al. "Shifts in growth strategies reflect tradeoffs in cellular economics." Molecular systems biology 5.1 (2009): 323.
Goelzer, Anne, Vincent Fromion, and Gérard Scorletti. "Cell design in bacteria as a convex optimization problem." Automatica 47.6 (2011): 1210-1218.
O'brien, Edward J., et al. "Genome‐scale models of metabolism and gene expression extend and refine growth phenotype prediction." Molecular systems biology 9.1 (2013): 693.


Line 44: existing contenders: none that I can see from the rest of your paper.

Line 47: which are the valuable insights? I don’t get that from the rest of the paper. This comes back to the 2nd major issue for me – lack of biological interpretation of your predictions.

Line 59: The models also allow for… (it is not models, but modeling paradigms which allow for this integration, and which ones do you refer to here? I guess GECKO and MOMENT, but then it might be nice to name them already)

Line 86-87: I think you have to mention other approaches that also predict protein concentrations (such as RBA and ME-models), even if later for some reason you don’t want to compare to them. Like this is just seems that you “ignore them”.

Lines 88-93: First you claim it is possible to calculate the optimal concentrations of enzymes, then that you can estimate kcat values, but you cannot get both simultaneously. What do you want to say by this sentence?

Lines 99-102: All of this (gene expression, regulatory pathways, metabolic flux) changes even if you move from a “suboptimal” to “optimal” condition. This is why I would want you to explain what you mean by optimal. The person who introduced the word stress to our current biological dictionary, Hans Seyle, called it any kind of change in environmental condition which caused to organism to need to adapt its internal milieu. No need for one condition to be “optimal”, other “suboptimal”.

Lines 106-107: It is finally stated that you need the reference state – this, in my opinion, belongs to the abstract.

Lines 110-111: You compare to an unpublished version of pFBA – not valid to call it a “competing method”. I would say that GECKO or MOMENT would theoretically be your competing methods.

… here I first skip to methods …

Line 300: How do you fit your Manhattan distance into an LP problem definition? Please under “min” indicate what are the decision variables of your LP problem.

Line 308: what do you mean by a biomass pseudo-reaction? Is it just a standard biomass reaction taken from the respective metabolic reconstructions you are using?

Line 311 & 313: Please explain how you transform the Euclidian distance measure to a quadratic programming problem. It can be done, but is not an obvious reformulation, and readers could benefit from it being explicit. Provide Q matrix and c vector. Also, nowhere in the text you mention quadratic programming – be explicit! 

--- comment on this entire part --- 
One great value of your paper could be the summarization and standardization of all of these experiments – I would emphasize this, and offer this data in an easily readable format that can be parsed by open software like Python (not proprietary like Matlab). I know this is a major effort and I think it’s great that you’ve done it, I guess a significant portion of your future citations could come from this if you make it easily accessible (and mention in somewhere earlier in the paper).

Line 333: what is the batch model?

Line 346: totally agree.

Line 355: Maybe you can indicate where exactly is this flexibilization described (in their paper or in the toolbox documentation). As a reader of your paper, this step seems very important to me and I would want to know in more detail the exact procedure by which this is done.

Line 375-380: I fully understand that you need a way to transform proteomics results into enzyme concentrations, but why don’t you use the actual measured individual enzyme concentrations in this, instead of just the total protein concentration? Why reduce the information content so drastically? Seems a bit pointless to have the individual measurements like this? 
I don’t see a plot which compares the predictions from your algorithm to actual proteomics? It is not enough to say you did the comparison, seeing the results of that comparison would be much better.
So – I would want to see the comparison between your baseline and actual proteomics.

--- back to Results ---


Line 127: it says see Methods, but when I see methods, I still don’t know what this flexibilization actually does.

Line 132: Did you choose the enzyme or? Why such big difference in the number of enzymes?

Line 135-136: This relates to my major issue No.1.

Line 140: You just introduced this term: the null model – what is it? The EsKcat version? What does it mean, kcat  values are used directly as the enzyme usage? You mean you get E = nu / kcat directly or what?

Lines 194 – 201: What are your two different scenarios? From what I understand, one is either 0 or 1, the other is from 0.1 to 1. But if that is the case, how can  the result of your first scenario be “with decreasing correlation values as lambda values increased”? Is it not bound to just 0 or 1 in that scenario? I don’t understand this paragraph.

Line 263: What do you mean by “cells are operating at saturation point for all metabolites”? I do agree with the rest of the things stated about this in the following text, I just don’t understand this phrasing.
