# Supplementary material for: PARROT: Prediction of enzyme abundances using protein-constrained metabolic models
Source: PLoS Comput Biol. 2023 Oct 19;19(10):e1011549. doi: 10.1371/journal.pcbi.1011549 (PMC10617714; doi:10.1371/journal.pcbi.1011549)
Supplement: Attachment — Submitted filename: Response_to_reviewers.docx [file pcbi.1011549.s009.docx]

**Response to the reviewers’ comments to manuscript PCOMPBIOL-D-23-00821**

**PARROT: Prediction of enzyme abundances using protein-constrained metabolic models**

Mauricio Alexander de Moura Ferreira^a^, Wendel Batista da Silveira^a^, Zoran Nikoloski ^b,c,*^

We have revised the manuscript and addressed the comments indicated by the reviewers. Detailed point-by-point improvements and answers (*in red*) to the reviewers’ comments are listed below.

# Reviewer 1

The authors present their method, PARROT, which employs constraint-based approaches for minimizing the enzyme allocations to assess the protein allocations in microorganisms growing under different suboptimal conditions. These constraint-based approaches attempt at optimizing the enzyme allocations rather than other contending approaches which rely on flux redistributions. The authors benchmarked the performance of their approach/es on two model microorganisms---*E. coli* and *S. cerevisiae*---and propose the use of protein-constrained genome-scale metabolic models (pcGEMs) for studying microbes in different conditions.

We appreciate the reviewer’s interest in our work and for the brief summary of our results.

## Major Issues:

1. Given that the growth of microorganisms in different conditions, particularly suboptimal conditions, is central to the positioning of this method and the manuscript, it is important that the authors provide some more motivation and context for the need to study the microbes is such varied conditions.

The authors can easily find suitable literature from antimicrobial resistance where microbes, like the superbug ESKAPE pathogens, evolve and continue to grow even under the effect of antibiotics---the suboptimal growth conditions. There are studies that have reported evidences of a microbial strain becoming resistant to an antibiotic under prolonged exposures.

- doi.org/10.3389/fmicb.2021.714284

- doi.org/10.1093/femsre/fux013

Another avenue for reference literature could be the clinical growth of microbial communities using selective enrichment media.

Thank you for this suggestion. Indeed, we agree that highlighting the biological importance and applicability of our method can be a compelling selling point for PARROT. We added a few sentences in the Introduction section and a paragraph in the Discussion section, as listed below:

Before the change – L102-104:

Despite the aforementioned advances in predicting protein abundances, the problem of predicting enzyme allocation under suboptimal growth conditions remains largely unexplored. […]

After the change:

Despite the aforementioned advances in predicting protein abundances, the problem of predicting enzyme allocation under suboptimal growth conditions remains largely unexplored. This could be useful to explore how cells adapt to changing environmental conditions, such as those faced by yeasts in industrial fermentations or by pathogenic bacteria when exposed to antibiotics.

Before the change – L216:

By comparing the predictions to a baseline constructed with experimental proteomics measurements for suboptimal conditions, […]

After the change:

Understanding how cells adjust enzyme allocation during growth conditions apart from the physiological optimum might prove useful to study, for example, the adaptability of yeasts when exposed to ethanol during fermentation. Ethanol hinders growth and enacts several changes to membrane structure and function, causes protein denaturing and metabolic imbalances [26]. The yeast *Kluyveromyces marxianus*, important for fermentation of dairy products, adapts to ethanol stress by strengthening the cell membrane by accumulating trehalose and by altering the content of ergosterol and unsaturated fatty acids in the cell membrane, along with changes in several regulatory pathways [27,28]. Another example is the resistance to antibiotics observed in human and animal bacterial pathogens. The prolonged and continuous exposure to antibiotics leads to a selective pressure where the bacterial population acquires resistance to the antibiotic which it is being exposed to, by means of mutations or through acquisition of mobile genetic elements, leading to the emergence of antibiotic-resistant strains [29]. For instance, antibiotic-resistant strains of *Klebsiella pneumoniae*, an ESKAPE pathogen, harbours many plasmids containing genes responsible for coding enzymes that break the antibiotic molecule. An example is the ampC gene family, which codes for β-lactamases, capable of degrading antibiotics such as penicillin, monobactams, cephalosporins and carbapenems. Resistance to these antibiotics is achieved by overexpression of these genes and overproduction of β-lactamases, along with the production of binding proteins that target the antibiotic molecules [30]. Overproduction of enzymes leads to disrupted metabolic states due to the inefficient allocation of resources [31], which can be exploited for therapeutic efforts such as enhancing antibiotic sensitivity [32].

By comparing the predictions to a baseline constructed with experimental proteomics measurements for suboptimal conditions, […]

2. The Results section should be rewritten to document the specifics and performance details of all approaches being compared/benchmarked. There are subsections titling PARROT to have outperformed other contending methods, but this is an inference to be made from the specific results. The exact specifics about the differences and similarities in results from different approaches are either not well documented/highlighted or are mentioned in the supplementary material. Therefore, from a reader's point of view, the actual results and performance of PARROT are not easy to follow and interpret.

Thanks for your suggestion. We have reorganized and rewritten some parts of the Results section to better explain the specifics and performance details of the approaches we present. The number of changes were too many to list in this document without making it too convoluted, but the changes can be seen in the marked-up version of the revised manuscript.

# Reviewer 2

This manuscript introduces a method for calculating Protein allocation Adjustment foR suboptimal enviROnmenTs (PARROT). With this method, the authors aim to study the metabolic phenotype of new, suboptimal conditions. The method is based on the principle of minimal adjustment of the proteome starting from a reference state. This is represented in the objective function which minimizes the distance between the reference condition and the suboptimal conditions. The authors compared different distance metrics (Manhattan and Euclidean distance) and the effect of adding the minimization of the flux distance to the objective functions. They found that for models of *Saccharomyces cerevisiae* and *Escherichia coli*, their method outperformed normal pFBA (minimizing the sum of all fluxes). Furthermore, they observed that the adding minimization of difference in flux distributions does not improve the results, even worsens it.

We would like to thank the reviewer for briefly summarizing the main points of our manuscript.

## Major comments

### General:

• Variants are used with different intentions (different conditions or different distance functions). This is confusing for the reader. Please be concise in how you refer to the different models or distance functions.

Thanks for your suggestion. We changed the way we refer to the variants to make it uniform and concise. Below we list the changes made to the manuscript:

Before the change – L24-27:

We demonstrate that the PARROT variant that minimizes the Manhattan distance of enzyme allocations outperforms existing approaches based on the parsimonious distribution of fluxes or enzymes for both *Escherichia coli* and *Saccharomyces cerevisiae*.

After the change:

We demonstrate that the PARROT variant that minimizes the Manhattan distance between the enzyme allocation of a reference and an alternative condition outperforms existing approaches based on the parsimonious distribution of fluxes or enzymes for both *Escherichia coli* and *Saccharomyces cerevisiae*.

Before the change – L171-191:

To further evaluate the predictions made by PARROT, we investigated how the usage of a baseline constructed by minimizing the second norm of the vector $\mathbf{E}_{\mathbf{s}}^{\mathbf{exp}}$ impacts the comparisons. To this end, we repeated all comparisons as performed for a baseline constructed by minimizing the first norm, using the predicted $\mathbf{E}_{\mathbf{s}}$ obtained by the PARROT variants. Importantly, the results were consistent between the two baseline approaches. For *S. cerevisiae*, the minimization of the weighted sum of the Manhattan distance of enzyme usage and Manhattan distance of flux distributions (LP2, Methods) was the variant that achieved the highest mean Pearson correlations than pFBA and its modified implementation (Figure S1). For the RMdSE, all PARROT variants had errors comparable to the positive controls (Figure S2). As observed for comparisons using the first norm baseline, all PARROT variants outperformed the null model.

The comparisons performed using predictions obtained for *E. coli* were also consistent with different variants of PARROT that outperformed pFBA. Considering the Pearson correlations, the minimization of the Manhattan distance (LP1, Methods) and the minimization of the weighted sum of the Manhattan distance of enzyme usage and Manhattan distance of flux distributions (LP2, Methods) also had the highest median correlations and were significantly different to pFBA. Likewise, these PARROT variants also had a significant difference to EsKcat, the modified implementation of pFBA (Figure S3). The comparison of RMdSE values were also consistent, as the errors were comparable to the positive controls (Figure S4). Altogether, these results highlight the robustness of estimations of $\mathbf{E}_{\mathbf{s}}$ obtained from PARROT.

After the change:

To further evaluate the predictions made by PARROT, we investigated if the construction of the baseline could impact the correlations. Thus, we reconstructed the baseline by minimizing the 2-norm of the vector $\mathbf{E}_{\mathbf{s}}^{\mathbf{exp}}$ instead of the 1-norm. To this end, we repeated all comparisons as performed for a baseline constructed by minimizing the 1-norm, using the predicted $\mathbf{E}_{\mathbf{s}}$ obtained by the PARROT variants. Importantly, the results were consistent between the two baseline approaches. For *S. cerevisiae*, the LP2 variant achieved the highest median Pearson correlations compared to pFBA and its modified implementation (Figure S1). For the RMdSE, all PARROT variants had errors comparable to the positive controls (Figure S2). As observed in the comparisons with the 1-norm baseline, all PARROT variants outperformed the null model.

The comparisons performed using predictions obtained for *E. coli* were also consistent with different variants of PARROT that outperformed pFBA. Considering the Pearson correlations, the LP1 and the LP2 variants also had the highest median correlations and were significantly different to pFBA. Likewise, these PARROT variants also showed significant difference to the modified implementation of pFBA (Figure S3). The comparison of RMdSE values were also consistent with this observation, as the errors were comparable to the positive controls (Figure S4). Altogether, these results highlight the robustness of estimations of $\mathbf{E}_{\mathbf{s}}$ obtained from PARROT.

Before the change – L223-230:

From the different variants of PARROT, the minimization of the Manhattan distance (LP1) and the minimization of the weighted sum of the Manhattan distance of enzyme usage and Manhattan distance of flux distributions (LP2) were the best contenders. The variants QP1 and QP2 – that minimizes Euclidean distances instead of Manhattan distances – resulted in good but also inconsistent performance between *S. cerevisiae* and *E. coli*. This agrees with the fact that the first norm distance is the natural metric for enzyme abundances in the cell, because a change in enzyme concentration requires ribosomal activity that scales linearly with the enzyme abundance [26].

After the change:

From the different variants of PARROT, LP1 (minimization of the Manhattan distance of enzyme usage distributions) and LP2 (the minimization of the weighted sum of the Manhattan distance of enzyme usage and Manhattan distance of flux distributions) were the best contenders across conditions for both *S. cerevisiae* and *E. coli*. The variant QP1 (minimization of the Euclidean distance of enzyme usage distributions) resulted in good, but inconsistent performance between *S. cerevisiae* and *E. coli*. For QP2 (the minimization of the weighted sum of the Euclidean distance of enzyme usage and Euclidean distance of flux distributions), it had poor results for *S. cerevisiae*, while having good results for *E. coli*, albeit worse than the other variants. This agrees with the fact that the first norm distance is the natural metric for enzyme abundances in the cell, because a change in enzyme concentration requires ribosomal activity that scales linearly with the enzyme abundance [33].

### Abstract:

• L. 21-22: In this sentence, you try to explain the main function of PARROT. I think this sentence is confusing for someone who hasn’t read the paper yet, since the meaning of ‘enzyme allocation’ and ‘enzyme allocation adjustment’ is unclear from the first part of the abstract. I recommend clarifying these terms or rephrasing to something a lay reader can understand.

Thanks for pointing this out. We have rewritten these sentences to make it clearer for the reader.

Before the change – L21-22:

Here we propose a family of constrained-based approaches, termed PARROT, to predict enzyme allocations based on the principle of minimizing the enzyme allocation adjustment using protein-constrained metabolic models.

After the change:

Here we propose a family of constrained-based approaches, termed PARROT, to predict how much of each enzyme is used based on the principle of minimizing the difference between a reference and an alternative growth condition using protein-constrained metabolic models.

• L 25: From the abstract it is unclear between what the distance is calculated. This could be easily added by replacing ‘of enzyme allocations’ with ‘between the allocation of enzymes of a reference and suboptimal condition.

Thanks for raising this issue. We reworded the sentence considering your suggestion.

Before the change – L24-27:

We demonstrate that the PARROT variant that minimizes the Manhattan distance of enzyme allocations outperforms existing approaches based on the parsimonious distribution of fluxes or enzymes for both *Escherichia coli* and *Saccharomyces cerevisiae*.

After the change:

We demonstrate that the PARROT variant that minimizes the Manhattan distance between the enzyme allocation of a reference and an alternative condition outperforms existing approaches based on the parsimonious distribution of fluxes or enzymes for both *Escherichia coli* and *Saccharomyces cerevisiae*.

### Materials and methods:

• In the code 5% flexibility is allowed on the growth rate, which is not described in the Materials and methods.

Thanks for mentioning this omission. We now describe this at the end of the section “Experimental data and simulation constraints”.

Before the change – L344-346:

In addition, we excluded the temperature stress conditions from Lahtvee et al. [24], as temperature can severely impact the function of enzymes [38], and temperature stress responses entail changes beyond metabolic flux redistribution [16].

After the change:

In addition, we excluded the temperature stress conditions from Lahtvee et al. [24], as temperature can severely impact the function of enzymes [50], and temperature stress responses entail changes beyond metabolic flux redistribution [16]. To prevent overconstraining the models, we allowed 5% flexibility on the growth rate. For other constraints, we allowed flexibility by increments of 1% until the measured growth rate is achieved.

• L. 357: It is not clear if 'values' refer to the original protein measurements or to the relaxed protein values.

Thanks for pointing this out. We reworded the sentence to make it clearer.

Before the change – L356-357:

We then used for the $\mathbf{E}_{\mathbf{ref}}$ vector of each experiment the values for flexibilised proteins along with values for proteins that were unchanged.

After the change:

For the $\mathbf{E}_{\mathbf{ref}}$ vector of each experiment, we then used these relaxed protein measurements while keeping the original values for proteins that were unchanged by allowing flexibility.

• L. 382: What does ‘The second norm in constructing a baseline’ mean in mathematical terms? This is not clear from the text and is important in interpreting this analysis.

Thank you for mentioning this. We intended to minimize the total enzyme allocation of the models integrated with experimental measurements for each growth condition, as done in Equation 11, but minimizing the L2 norm of $\mathbf{E}_{\mathbf{s}}^{\mathbf{exp}}$ instead of the L1 norm. We rewrote the sentence to make it clearer, along with adding the objective function of this problem. The subsequent equations were renumbered to adjust for the inclusion of a new one.

Before the change – L381-382:

We also performed a robustness analysis to check the effect of using the minimization of the second norm in constructing a baseline.

After the change:

We also performed a robustness analysis by checking the effect of minimizing the 2-norm of $\mathbf{E}_{\mathbf{s}}^{\mathbf{exp}}$ to construct the baseline, instead of the 1-norm, keeping the constraints defined in Equations 12-16:

$\min\left\| \mathbf{E}_{\mathbf{s}}^{\mathbf{exp}} \right\|_{2}$	(17)

### Results:

• L. 132: ‘18-336’ and L. 156: ‘19-141’. Why is the range of number of enzymes in the predicted suboptimal conditions so wide? How does this influence the interpretation of the results? I can imagine that the number of predictions influence the value of the accuracy metrics.

Thanks for bringing attention to this. The range is wide because of a few outliers, as it considers the number of enzymes predicted across all experimental conditions and across all minimization problems. More specifically, the low number of predicted enzymes come from the QP2 variant of PARROT which, as discussed in the manuscript, is the variant with the worst performance. For the other variants (LP1, LP2 and QP1), the number of predicted enzymes is all on the higher end (around 300 for *S. cerevisiae*, around 140 for *E. coli*). We have reorganized and rewritten some parts of the Results section and now we present the results of each variant of PARROT individually. The number of changes to the Results were too many to list in this document without making it convoluted, but the changes can be seen in the marked-up version of the revised manuscript.

• L. 140-141: Here you mention that the kcat values are used as direct measure of the enzyme usage. What does this mean? Do you mean that you use the values of the enzyme variables as enzyme concentrations? I miss a description of this validation method in the ‘Methods’ section.

Thanks for mentioning this. We used this as a negative control by considering that $\mathbf{E}_{\mathbf{s}}=k_{cat}$, since both $\mathbf{E}_{\mathbf{exp}}=\mathbf{v}_{\mathbf{exp}}\cdot k_{cat}$ and $\mathbf{E}_{\mathbf{s}}=\mathbf{v}_{\mathbf{s}}\cdot k_{cat}$ hold. We wanted to check whether some of the correlation between $\mathbf{E}_{\mathbf{exp}}$ and $\mathbf{E}_{\mathbf{s}}$ could be attributed directly to $k_{cat}$. Ideally, PARROT would outperform this negative control, attributing the correlations to $\mathbf{v}_{\mathbf{exp}}$ and $\mathbf{v}_{\mathbf{s}}$, which are predicted by PARROT. We did briefly describe this validation method in the Methods section, between lines 398 and 403. We have expanded the explanation in both sections to make it clearer for the reader.

Before the change – L140-141:

Further, all PARROT variants outperformed the null model, where $k_{cat}$ values are used directly as the enzyme usage.

After the change:

This variant also outperformed the null model, where $k_{cat}$ values are used directly as the enzyme usage ($E_{s,i}=k_{cat}^{ij}$). This negative control is useful to determine the contribution of $k_{cat}$ values to the correlation between $\mathbf{E}_{\mathbf{exp}}$ and $\mathbf{E}_{\mathbf{s}}$ (see Methods).

Before the change – L398-403:

Lastly, as a negative control to benchmark the performance of PARROT, we equated $E_{s,i}$ to $k_{cat}^{ij}$, meaning that $k_{cat}$ values we used directly as the enzyme usage. We calculated the correlation values and RMdSE for all assessed optimization problems and compared them to the predictions of pFBA and its modified implementation using a Pairwise Wilcoxon rank sum test with Bonferroni correction.

After the change:

Lastly, as a negative control to benchmark the performance of PARROT, we equated $E_{s,i}$ to $k_{cat}^{ij}$ ($E_{s,i}=k_{cat}^{ij}$), meaning that $k_{cat}$ values we used directly as the enzyme usage. This allows for determining how much of the correlation between $\mathbf{E}_{\mathbf{exp}}$ and $\mathbf{E}_{\mathbf{s}}$ can be attributed directly to $k_{cat}$ values.

• l. 153: Why did you choose the smallest dilution rate as the reference condition? What is the motivation for this?

Thanks for your question. We chose the smallest dilution rate as the reference condition because the low dilution rate ensures that cells are growing aerobically and with a stable phenotype. At higher dilution rates, many species undergo metabolic shifts from a respiratory metabolism to a fermentative metabolism, termed overflow metabolism (or the Crabtree effect, in yeasts). Additionally, as $\mu$ increases, so does the need for additional nutrient transporters, given that the concentration of the limiting nutrient gradually increases as well. Thus, for a chemostat experiment dealing with increasing dilution rates, it does not make sense to use higher dilution rates as a reference point, given the observed changes in cell physiology. We expanded the explanation in the manuscript, so it is clearer for readers.

Before the change – L151-154:

Further, as reference condition we considered the control samples or the chemostat measurements with the smallest dilution rate (Table S2). The number of enzymes contained i**n** $\mathbf{E}_{\mathbf{ref}}$ ranged from 152 to 188 depending on the control experimented used.

After the change:

Further, as reference condition we considered the control samples or the chemostat measurements with the smallest dilution rate (Table S2). We chose the smallest dilution rate to ensure that cells are growing aerobically and to prevent the metabolic shifts seen in higher dilution rates (e.g., overflow metabolism). The number of enzymes contained in $\mathbf{E}_{\mathbf{ref}}$ ranged from 152 to 188 depending on the control experimented used.

• Figure 2, 3, S2 and S3: These type of plots do not give a FAIR visualization of the results. Due to the scale of the plot, the distribution of the data in each box is impaired. I suggest adjusting the scale and trying out different ways of data visualization, such as a violin plot with individual data-points or even a raincloud plot.

Thanks for your suggestion. We changed the box plots to violin plots.

• L. 189: Consistent with what? You can clarify this by adding ‘with this observation’ after ‘consistent’.

Thanks for pointing this out. We corrected the sentence considering your suggestion.

Before the change – L189:

The comparison of RMdSE values were also consistent.

After the change:

The comparison of RMdSE values were also consistent with this observation.

### Discussion:

• L. 199-202 and 241-255: In the Results you describe the effect of alternating lambda on the occurrence of higher/lower Pearson correlation values. Later, in the Discussion you explain the meaning of a lambda value of 1 or 0. This part contains sufficient technical explanations, but I miss the biological explanation of this factor. What mechanism does this represent? What did you learn about Biology from this result?

Thanks for raising this issue. We expanded the biological explanation in the Discussion section. Using the parameter $\lambda$ , we tried to check how much the flux distribution contributes to predicting enzyme usage. The mechanism corresponds to the knowledge that under changing environments, cells adopt a strategy to first change gene expression, leading to changes in protein allocation. By doing this, metabolic flux is set to change as a consequence of the first two modifications (Yang et al. 2023, DOI: 10.1038/s41467-023-37930-x). Given that metabolic flux can be defined as function of enzyme concentration and its catalytic efficiency to a particular substrate (i.e., $v=k_{cat}\cdot[E]$), changes in metabolic flux under a changing environment occurs after the proteome has changed. Using higher $\lambda$ values in the objective function of LP2 and QP2 forces the model to optimize for both the enzyme usage distribution and the flux distribution, as if both processes occurred simultaneously in the cell. Lower $\lambda$ values allow for the model to prioritize the minimization of the enzyme usage distribution, being close to the biological reality. By setting a $\lambda$ of 0, metabolic fluxes are not considered at all, leaving the model free to solve for minimal enzyme usage redistribution, and calculating the flux distribution as a function of enzyme usage, as is observed in the cell (Chen et al. 2023, DOI: 10.1073/pnas.2302779120). This supports the conclusion that minimizing the readjustment of enzyme resource allocation is one principle underpinning microbial adjustment to an alternative (non-reference) condition.

Before the change – L241-258:

The parameter $\lambda$ is a factor that weights the usage of metabolic fluxes for the optimisation problem. By varying this value between 0 and 1, we could assess how much the minimization of metabolic fluxes contributes to the problem of predicting enzyme usage. A $\lambda$ value of 0 would render the variants LP2 and QP2 equivalent to LP1 and QP1, respectively, as metabolic flux would be neglected in the optimal solutions. A $\lambda$ value of 1, in the other hand, renders LP2 and QP2 as equivalent to using a pcGEM with the canonical implementation of MOMA, which considers all fluxes equally. When the two PARROT variants are free to vary $\lambda$ between 0 and 1, there is a strong preference for lower $\lambda$ values. When constraining $\lambda$ to a value between 0.1 and 1, higher values of $\lambda$ are present but still not more prevalent than lower values of $\lambda$. This suggests that the joint minimization of fluxes and enzymes is not a principle of flux redistribution, and the principle is guided by minimization of resource redistribution, as best captured by LP1 and QP1, and by LP2 and QP2 with low values of $\lambda$. Thus, by being proteome-aware, PARROT is better suited for simulations using pcGEMs than the quadratic and linear implementations of MOMA, given that higher participation of metabolic fluxes lowers the overall predictive performance. Altogether, we demonstrated that minimizing the readjustment of enzyme resource allocation is one principle underpinning microbial adjustment to a suboptimal condition. Thus, PARROT may allow for study and engineering of microbial cell factories, as these are often under suboptimal growth conditions in industrial settings [28].

After the change:

The parameter $\lambda$ is a factor that weights the usage of metabolic fluxes for the optimisation problem. By varying this value between 0 and 1, we could assess how much the minimization of metabolic fluxes contributes to the problem of predicting enzyme usage. A $\lambda$ value of 0 would render the variants LP2 and QP2 equivalent to LP1 and QP1, respectively, as metabolic flux would be neglected in the optimal solutions. A $\lambda$ value of 1, in the other hand, renders LP2 and QP2 as equivalent to using a pcGEM with the canonical implementation of MOMA, which considers all fluxes equally. When the two PARROT variants are free to vary $\lambda$ between 0 and 1, the optimum is reached for lower $\lambda$ values. This can be explained by the experimental observation that in changing environments, cells adopt a strategy of initially adjusting gene expression, which subsequently results in shifts in protein allocation. Consequently, this leads to subsequent changes in metabolic flux [20,35]. When constraining $\lambda$ to a value between 0.1 and 1, higher values of $\lambda$ are present but still not more prevalent than lower values of $\lambda$. This suggests that the joint minimization of fluxes and enzymes is not a principle of flux redistribution, given that when higher λ values are employed in the objective function of LP2 and QP2, the model simultaneously optimizes both the enzyme usage distribution and the flux distribution, as though the cell performs these processes at the same time. Instead, the principle is guided by minimization of resource redistribution, as best captured by LP1 and QP1, and by LP2 and QP2 with low values of $\lambda$, from which a flux redistribution is then later derived. Using lower λ values, the model prioritizes minimizing the enzyme usage distribution, aligning more closely with experimental observations. With a λ of 0, the model disregards metabolic fluxes entirely, enabling it to focus on solving for minimal enzyme usage redistribution, only calculating the flux distribution as a function of the former, mirroring what is observed in the cell [36]. Thus, by being proteome-aware, PARROT is better suited for simulations using pcGEMs than the quadratic and linear implementations of MOMA, given that higher participation of metabolic fluxes lowers the overall predictive performance. Altogether, we demonstrated that minimizing the readjustment of enzyme resource allocation is one principle underpinning microbial adjustment to an alternative condition, aligning with experimental evidence. Thus, PARROT may allow for study and engineering of microbial cell factories, as these are often under suboptimal growth conditions in industrial settings [37].

• L. 280: ‘underestimating capacity’ I think capacity is not the correct word here. Capacity is a feature, not a result. I would use ‘the underestimation of protein concentrations’ instead.

Thanks for your suggestion. We adjusted the sentence accordingly.

Before the change – L280:

Nevertheless, other approaches for estimating *in vivo* protein concentrations would still need to overcome the underestimating capacity of pcGEMs […]

After the change:

Nevertheless, other approaches for estimating *in vivo* protein concentrations would still need to overcome the underestimation of protein concentrations of pcGEMs […]

• L. 281: Another approach is to specifically consider un/under-used enzymes as a separate protein sector as done in Alter et al. (2021) (https://doi.org/10.1128/msystems.00625-20). Consider discussing/citing this source.

Thanks for your suggestion. We briefly discuss the idea of using proteome sectors, as we cite in Mori et al. (2017, DOI: 10.1038/s41467-017-01242-8). The idea of considering unused and/or underused enzymes is interesting, and we hint at the end of the Discussion section a possible next step in the development of pcGEMs, which is to overcome the underestimation of protein concentrations and to consider the proteome reserve in the problem formulation. This protein reserve contains the enzymes which are not catalytically active, which is exactly what the unused protein sector proposed by Alter et al. (2021) refers to. We added some sentences in the Discussion section to discuss this reference.

Before the change – L279-283:

Nevertheless, other approaches for estimating *in vivo* protein concentrations would still need to overcome the underestimating capacity of pcGEMs, especially by considering the proteome reserve. These approaches could include features such as cellular machinery beyond enzymes that participate in metabolism, or by integrating constraint-based approaches with data-driven approaches.

After the change:

Nevertheless, other approaches for estimating *in vivo* protein concentrations would still need to overcome the underestimation of protein concentrations of pcGEMs, especially by considering the proteome reserve. Interestingly, Alter et al. [41] deal with the problem of catalytically inactive enzymes by considering a protein sector of unused enzymes, along with active enzymes sector and the translational protein sectors. The unused protein sector, $\phi_{UE}$, is calculated by relating the decrease of the concentration of unused enzymes to the increase in the substrate uptake rate. This allows for the model to predict the adaptability of *E. coli* to changing environmental conditions. However, the initial value $\phi_{UE,0}$, which is the unused enzyme concentration when the substrate uptake is zero, is obtained from simulations using a ME model, which are notoriously difficult to parameterize [42]. New approaches could include features such as cellular machinery beyond enzymes that participate in metabolism, or by integrating constraint-based approaches with data-driven approaches, as recently done in the CAMEL approach [43].

## Additional minor comments:

### **General:**

The use of ‘suboptimal’ as term for the condition which is not the reference condition is questionable. For example, in the chemostat cultures, you choose the lower growth rate as reference condition, making the other conditions thus ‘suboptimal‘. In terms of biology this is incorrect: the cells are growing even faster in these conditions. I would choose a word as ‘alternative‘ condition instead.

Thanks for your suggestion. We agree that using “alternative” instead of “suboptimal” would be more inclusive for the different conditions we use, and we have changed all mentions of “optimal” to “reference”, and “suboptimal” to “alternative”, including in the acronym. The number of changes were too many to list in this document but can be seen in the marked-up version of the revised manuscript. It is important to point out that it is also biologically incorrect to assume that cells achieving high growth rates means that the cells are “growing faster” (as this is not what $\mu$ represents, but rather it is best represented by the doubling time), or that cells “growing faster” means that culture conditions are necessarily optimal or better than at a low growth rate, as doubling time varies not only with environment, but is also largely dependent on strain and on species. This is true even for *E. coli*: as we have mentioned earlier, *E. coli* undergoes overflow metabolism at high growth rates, that is, acetate overflow, along with many other physiological changes and adaptations to sustains the high growth rate that may not necessarily be considered “optimal”.

l. 21, l. 294: I think the term ‘enzyme allocations’ is confusing, as allocation is not commonly used in plural. Perhaps 'the allocation of enzymes' suits better here?

Thanks for your suggestion. We’ve changed the sentences accordingly.

Before the change – L20-21:

Here we propose a family of constrained-based approaches, termed PARROT, to predict enzyme allocations based on the principle […]

After the change:

Here we propose a family of constrained-based approaches, termed PARROT, to predict how much of each enzyme is used based on the principle […]

Before the change – L293-295:

We define and compare four different objectives to model the distance between enzyme allocations in suboptimal and reference states:

After the change:

We define and compare four different objectives to model the distance between the allocation of enzymes in alternative and reference states:

l. 178: ‘than’ should be ‘compared to’

Thanks for pointing this out. We adjusted the sentence.

Before the change – L178:

[…] highest mean Pearson correlations than pFBA and its modified implementation (Figure S1). […]

After the change:

[…] highest mean Pearson correlations compared to pFBA and its modified implementation (Figure S1) […]

l.180: The following sentence: ‘As observed for comparisons using the first norm baseline’ could be replaced by: ‘As observed in the comparisons with the first norm baseline’. In my opinion, this would clarify the message of what is compared with what.

Thanks for your suggestion. We replaced the sentence.

Before the change – L180:

As observed for comparisons using the first norm baseline, all PARROT variants outperformed the null model.

After the change:

As observed in the comparisons with the 1-norm baseline, all PARROT variants outperformed the null model.

l. 233: ‘using’ should be ‘with’.

Thanks for suggesting this change. We adapted the sentence.

Before the change – L233:

In constraining the pcGEMs using the proteomics measurements […]

After the change:

In constraining the pcGEMs with the proteomics measurements […]

l. 266-267: ‘is that cell overexpress’ could be replaced by ‘cells overexpress’.

Thank you for the suggestion. We adjusted the sentence accordingly.

Before the change – L266-267:

As noted by [30], however, is that cell overexpress enzymes and uses metabolite concentrations to control metabolic flux.

After the change:

As noted by [30], however, cell overexpress enzymes and uses metabolite concentrations to control metabolic flux.

l. 293: ‘associated flux state’ A flux state cannot be performed, as it is a state. You can replace part of this sentence with: ‘to perform growth and to maintain the associated flux state’ or ‘for growth and the associated flux state’.

Thanks for the correction. We replaced that part of the sentence.

Before the change – L292-293:

This is consistent with observations that micro-organisms minimize expenditures to perform a growth and associated flux state [25].

After the change:

This is consistent with observations that micro-organisms minimize expenditures to perform to perform growth and to maintain the associated flux state [25].

l. 355, 357, 574: 'to flexibilise’ is not an English verb. You can replace this by ‘to allow flexibility in the enzyme usage constraints' or 'relaxing the enzyme usage constraints'.

Thanks for the suggestion, but “to flexibilise” is an actual English verb. It is defined in the Merriam-Webster dictionary (<https://www.merriam-webster.com/dictionary/flexibilize>), albeit using the American spelling. However, we recognize that it is not very commonly used and may cause confusion to readers. Thus, we adjusted the sentence according to your suggestion.

Before the change – L354-355:

The GECKO Toolbox 2 identifies the enzyme usage values that most limit growth and flexibilises the values […]

After the change:

The GECKO Toolbox 2 identifies the enzyme usage values that most limit growth and allow flexibility in the enzyme usage constraints […]

Before the change – L356-357:

We then used for the $\mathbf{E}_{\mathbf{ref}}$ vector of each experiment the values for flexibilised proteins along with values for proteins that were unchanged.

After the change:

For the $\mathbf{E}_{\mathbf{ref}}$ vector of each experiment, we then used these relaxed protein measurements, while keeping the original values for proteins that were unchanged by allowing flexibility.

Before the change – L573-574:

The proteomics data from the reference state is pre-processed by integrating the data in a pcGEM using the GECKO Toolbox 2 and flexibilising its values.

After the change:

The proteomics data from the reference state is pre-processed by integrating the data in a pcGEM using the GECKO Toolbox 2 and allowing flexibility in its values.

### Code on GitHub:

`ptotREF = 0.61;

f_REF = 0.4;

sigma_REF = 0.4;

f_STR = 0.4;

sigma_STR = 0.4;`

Where do these values originate from and what do they represent? Please include comments or documentation to interpret these values. What is the source of these numbers (or the assumptions made to define them)?

Thanks for pointing out this omission. We have included comments in the code explaining the meaning of these values. The parameter *ptotREF* comes from the total protein content of the reference condition. The parameters f_REF and f_STR are the mass fraction of enzymes that are accounted for in the reference and alternative condition models, respectively. Finally, the parameters sigma_REF and sigma_STR are the average *in vivo* saturation of all enzymes in the reference and alternative condition models, respectively. These parameters are required for calculating the total enzyme usage, $E_{tot}$, which is then used to constrain the upper bound of the protein pool exchange pseudo-reaction**.** While ptotREF was obtained from experimental data, the other parameters were unfortunately not measured. As done by Domenzain et al (2022, DOI: 10.1038/s41467-022-31421-1), a default of 0.5 is used for *S. cerevisiae* when these values are not available for the growth condition being simulated. However, the default value of 0.5 resulted in $E_{tot}$ values lower than the required for growth, leading to overconstrained models. We adopted 0.4 as the default because this was the value that allowed growth while ensuring that the protein pool exchange pseudo-reaction was adequately constrained.

Using a dummy example on for example a core model to get to know the programming interface would be appreciated.

We appreciated your suggestion. We added some examples to better understand how to use PARROT.

# Reviewer 3

## Major issue:

1. This submission claims to outperform the “competing algorithms” in predicting enzyme abundances, but does not actually compare to any software which in the original publication claims to predict enzyme abundances. It only compares its results to pFBA, which is a method that predicts fluxes. The authors extend this version to predict enzyme abundances, but this is so-far unpublished method which no-one claims can predict enzyme abundances.

Thanks for raising this point, we expanded on the usage of pFBA and its modified implementation. Indeed, we compared PARROT to pFBA and a modified implementation where we change how the objective function is defined. As we mention during the text, methods for predicting protein abundance are mostly data-based, using machine learning or other statistical methods. So far, we are not aware of any constraint-based method that have been developed specifically for the problem of predicting protein abundance on a per-protein basis. There are a few methods that can predict protein abundance, but these are not tailored for this problem specifically: models of metabolism and macromolecular expression (ME-models) and resource balance analysis models (RBA models) are notoriously complex and require extensive amounts of data for parameterization, which at best only exists for a few microbial model species. Using these models for developing PARROT would have limited its application immensely. GECKO models, on the other hand, are relatively simple to reconstruct and to use for simulating phenotypes. The implementation of protein constraints in GECKO considers enzymes as “metabolites”, and enzyme usage happens as exchange pseudo-reactions using “fluxes”, which allows it to run on any platform such as COBRA or RAVEN. Given its implementation, the prediction of the enzyme usage distribution using GECKO models is performed in the same way as for the flux distribution, given that the former is a subset of the latter.

Regarding the pFBA, its initial intention in the original manuscript (Segre et al. 2002, DOI: 10.1073/pnas.23234939) was exactly to minimize the total necessary enzyme mass to find the optimal solution. It is able to predict fluxes in good accordance with fluxes predicted by GEMs integrated with transcriptomics and proteomics data. As we have done in this manuscript, other people have also used pFBA for comparisons in proposed new methods, such as the work of Jenior et al. (2020, DOI: 10.1371/journal.pcbi.1007099), which compared pFBA to an expanded implementation that uses transcriptomics data. Even the original authors of the GECKO method have used pFBA to predict enzyme usage (Domenzain et al., 2022, Figure 4D, DOI: 10.1038/s41467-022-31421-1). Therefore, it is reasonable to use pFBA as a benchmark and/or a positive control, given its ability to predict fluxes that match those of GEMs integrated with omics data (which is the case for PARROT). The case for our modified implementation (which we refer in the manuscript as EsKcat) is the same. The objective function of pFBA is defined to minimize the total sum of fluxes through an irreversible model, while the objective function of our modified implementation minimizes the product of $k_{cat}$ values and enzyme usage values, which equates to fluxes according to the constraint added in GECKO models: $v_{j}=k_{cat}^{ij}\cdot[E_{i}]$.

Further, we add that we have compared our method to MOMA, which is a similar approach that minimizes the Euclidean distance of flux distributions of a wild-type strain and a mutant strain. As we discuss in the manuscript, the implementation of the LP2 and QP2 variants using a lambda value of 1 is mathematically identical to the implementation of MOMA available in the COBRA Toolbox. By comparing the predictions of the LP1 and QP1 variants to LP2 and QP2 with a lambda value of 1, we see that LP1 and QP1 perform better. Therefore, using a method that focuses on the enzyme usage distribution is more suited to predict enzyme usage than a method that consider the whole flux distribution.

Before the change – L381-385:

We also performed a robustness analysis to check the effect of using the minimization of the second norm in constructing a baseline. In addition, we compared the predictions of our approaches to those obtained using an extension of parsimonious enzyme usage FBA (pFBA) [39] to consider enzyme constraints. To this end, for each sample of each experiment, we defined the optimization problem as: […]

After the change:

We compared the predictions of our approaches to those obtained using an extension of parsimonious enzyme usage FBA (pFBA) [39] to consider enzyme constraints. It is suitable for benchmarking our approach given its ability to predict phenotypes in good accordance with other methods that rely on transcriptomics and proteomics data. To this end, for each sample of each experiment, we defined the optimization problem as: […]

2. Lack of comparison to experimental data, and a biological analysis: do you see a particular metabolic adaptation that you would have missed by using pFBA alone? Where is the added value? Even a single example would suffice.

Thanks for raising this issue, and we stress that we actually do compare our results to experimental data, that is how all the correlations and error metrics are calculated. This is described in the section “Assessment of predicted enzyme usage distributions” of the Material and Methods. The experimental data is used to construct the baseline models, which we use as a comparison point for our predictions. Given that the baseline model represents the best-case scenario where the predictions of the pcGEM are matching to proteomics, it is fair to assume that the closer we get to the baseline, the better the model is and the closer the predictions get to biological reality.

## Minor (but important) issues:

2. The Manhattan distance method, which the authors claim is the best performing one, is formalized directly as an LP (linear programming) problem. To my knowledge, absolute values cannot be directly put into a formulation of an LP problem. I would expect a further elaboration on this – how did you transform it to an actual LP problem?

Thanks for your question. We transformed it to an LP problem by doing the following: first, we construct a delta model where we concatenate the two pcGEMs (the reference condition and the alternative condition), including the stoichiometric matrix, objective vector, upper and lower bounds vectors, and the RHS (zeroes) vector. We introduce two variables, delta+ and delta-, encoded in the objective vector, that represents the differences in the enzyme usage distribution between the two models. We define the objective function to be the minimization of the sum of delta+ and delta-. This implementation follows the linear implementation of MOMA in the COBRA Toolbox (Segre et al. 2002, DOI: 10.1073/pnas.23234939), but focusing on the enzyme usage distribution vector instead of the entire flux distribution vector. In addition, this is a standard transformation of 1-norm into a canonical linear program.

3. The use of term “optimal growth condition” is vague. I would appreciate authors either defining what they mean by optimal. Comparing the usage of that term to the rest of their text, it would seem to me that optimal actually means “the control condition”.

One simple example: it can be that the control condition for the growth of *E. coli* is at 37 degrees Celsius, and the “suboptimal” condition is growth at 42 deg C (Schmidt data), but the cells actually grow faster at the “suboptimal” growth condition.

We appreciate this issue. We agree that using “alternative” instead of “suboptimal” would be more inclusive for the different conditions we use, and we have changed all mentions of “optimal” to “reference”, and “suboptimal” to “alternative”, including in the acronym. The number of changes were too many to list in this document but can be seen in the marked-up version of the revised manuscript. It is important to point out that it is biologically incorrect to assume that cells achieving high growth rates means that the cells are “growing faster” (as this is not what $\mu$ represents, but rather it is best represented by the doubling time), or that cells “growing faster” means that culture conditions are necessarily optimal or better than at a low growth rate, as doubling time varies not only with environment, but is also largely dependent on strain and on species. This is true even for *E. coli*: as we’ve mentioned earlier for Reviewer 2, it undergoes overflow metabolism at high growth rates (more specifically, acetate overflow), along with many other physiological changes and adaptations to sustains the high growth rate that may not necessarily be considered “optimal”.

4. Lack of information how you go from proteomics to enzyme abundances (is this what you use GECKO for?) It wasn’t completely clear to me. Protein and enzyme abundances are quite different things… I guess this is what you use “flexibilization” for, but I’d appreciate an explanation on what that actually means, since you never compare to proteomics directly, but always to this “flexibilized” baseline. Why this strange baseline?

Thank you for pointing this out. We go from proteomics measurements to enzyme usage by constructing the baseline. First, we integrate the proteomics measurements into the pcGEMs using GECKO. For each enzyme in the pcGEM, the upper bound of the exchange pseudo-reaction is defined by the measurement obtained from proteomics. This value is converted from the unit of molecules/cell to mmol/g cell dry weight/h^-1^, which is the unit also used for metabolic fluxes. Regarding flexibilization, it is performed on the upper bounds of the exchange pseudo-reactions by identifying the values that limit growth and then relaxing these constraints until growth is achieved. The baseline itself is not “flexibilized”, it is the resulting enzyme usage distribution obtained after allowing flexibility on a few needed enzymes. To make it clear, the experimental data is there; it is just used in a different format to accommodate the requirements of a pcGEM. All the information is still there. This is necessary because there is a disparity between the enzyme usage measured by proteomics and the enzyme usage of enzymes that are actually carrying flux inside the cell, meaning that there are more enzymes than needed (see Yang et al., 2016 for an explanation on unused/underused enzymes, DOI: 10.1038/srep36734). The limitation of pcGEMs is that it can only predict the latter, meaning that comparing the predictions of PARROT directly to proteomics measurements is not optimal, since many enzymes have their usage values changed. The usage of a baseline is not “strange”, but a commonplace and often required method to assess how the predictions of a proposed model compares to a certain scenario. Given that the baseline model represents the best-case scenario where the predictions of the pcGEM are matching to proteomics, it is fair to assume that the closer we get to the baseline, the better our models are.

5. Table with parameters (P_tots, growth rates, uptake rates). Some things are in the supplementary (growth rates), and P_tots are in your MATLAB code, but one table summarizing this could be of great value to future researchers. Also – could you tell me where in Schmidt data did you find total protein content – I remember this being quite hard to find and existent only for one condition – or am I wrong here?

Thanks for your suggestion. There are two spreadsheets in the GitHub repository containing all the parameters. Regarding the total protein content, we referred to Schmidt by mistake and the data actually comes from Valgepea et al. (2013, DOI: 10.1039/C3MB70119K). Thanks for pointing out this mistake. It indeed is only available for one condition, which was the condition we chose as the reference for one group of experiments. We had to use this value for the other conditions, given it was the only one available. For *S. cerevisiae*, more data was available, which we of course have used.

## On software:

There are no clear instructions on how to run the software on the author’s GitHub pages. I have downloaded and installed Cobra, Raven and Gecko, and am still unable to run their code.

We appreciate you raising this issue. We have included some examples in the GitHub repository.

The mistake I get is that there is no method called “FlexibilizeConstraints” in none of these packages (including PARROT). I have recursively “grepped” all of these directories searching for “def FlexibilizeFluxes” to find a file in which this has been defined, but unsuccessfully.

Thanks for pointing out this omission. This function was supposed to be included in the repository; it mistakenly was not uploaded. We have uploaded it to the repository and the code now works as intended.

I would (first) want to be able to run the code, and (second) to have a much more informative README file which informs me of an example run (a code which should work).

We appreciate you raising this issue. Besides expanding the instructions to run the code, we provide two examples on how to run PARROT.

## What I see as options:

- either compare your results to a published algorithm which claims to predict enzyme abundances

- or weaken the claim about “outperforming the competition” (which in your current formulation is not exactly true) and provide more information about how your predictions compare to experimental data.

I believe we have clarified what we mean by “outperforming contending methods”. It is true given the boundaries we set and the comparisons we do. We would be more than happy to compare our results to a constraint-based method that claims to predict protein abundance, but outside pFBA and MOMA, unfortunately there have not been many contenders.

To be clear – I do see something valuable and interesting in your approach, but I do not see that you have proven your claims about “outperforming the competition”.
