# Supplementary material for: PARROT: Prediction of enzyme abundances using protein-constrained metabolic models
Source: PLoS Comput Biol. 2023 Oct 19;19(10):e1011549. doi: 10.1371/journal.pcbi.1011549 (PMC10617714; doi:10.1371/journal.pcbi.1011549)
Supplement: Attachment — Submitted filename: Response_to_reviewers.docx [file pcbi.1011549.s010.docx]

**Response to the reviewers’ comments to manuscript PCOMPBIOL-D-23-00821**

**PARROT: Prediction of enzyme abundances using protein-constrained metabolic models**

Mauricio Alexander de Moura Ferreira^a^, Wendel Batista da Silveira^a^, Zoran Nikoloski ^b,c,*^

We have revised the manuscript and addressed the comments indicated by the reviewers. Detailed point-by-point improvements and answers (*in red*) to the reviewers’ comments are listed below.

# Reviewer 1

Reviewer #1: I appreciate the authors for their efforts in updating the manuscript to incorporate the reviewers' feedback. I believe it has improved the manuscript substantially.

Having said that, there are still a few concerns that need to be addressed before this manuscript is accepted for publication:

We appreciate the reviewer’s recognition of our efforts in updating the manuscript.

1. Following the mentions from the title and the abstract section, the initial paragraphs of the introduction section should describe the need and utility of enzyme abundances and protein allocation, followed by subsequent literature on the challenges and advances in doing so. The current introduction starts with GEMs and protein-constraint GEMs and does not mention much about the need for enzyme abundances/protein allocations, with an underlying assumption that the reader already knows about the latter. It is recommended that the authors rethink and rewrite the initial paragraphs of the Introduction such that the context and scope of the manuscript are set for the readers without such major assumptions on the reader's part.

Additionally, some sentences have been worded in a bit convoluted manner while some other sentence combinations do not have a grammatical concordance that is easier to follow. For instance, the second sentence of the Abstract reads "Therefore, different experimental and machine learning approaches have been developed to quantify and predict protein abundances, respectively". The sentence structure suggests that it is contextual reasoning provided for what it described in the previous sentence. However, the previous sentence describes 'protein allocation', which is not mentioned in the following sentence at all. Such paraphrasing makes it quite difficult for a reader to follow the text. It is recommended that the authors proofread their manuscript with an end-reader in mind, preferably reviewing it with an English language expert, so that the presented content is easier to follow.

Thanks for raising these concerns and for the suggestions. We added a few sentences in the Introduction section to better highlight the importance of enzyme abundance and allocation. We also rephrased the mentioned sentence to make it clearer.

Regarding the importance of enzyme abundance, we do state that enzyme abundance is important for parameterizing protein constrained GEMs, along with catalytic rates (lines 56-57, 64-67), and that is the whole underlying concept behind our manuscript. We also clearly state the challenges and advances in doing this (lines 70-97). We start the introduction with GEMs because it is paramount to contextualize where the gap in knowledge is and where this kind of data is needed. Although we understand the need for better explaining the importance of enzyme abundance data, we should be careful not to mislead the reader into thinking PARROT can predict the abundance of *all* proteins and/or enzymes in a cell, which is not the case as we focus on metabolic enzymes integrated into pcGEMs. To predict quantitative proteomics data for all enzymes would be a daunting task, which would require approaches other than constraint-based modelling. Suggesting this idea to the reader would be thus highly misleading.

Before the change – L54-61:

While already useful for predicting a wide range of phenotypes, the predictive performance of GEMs has been further improved by integrating protein constraints, such as: enzyme catalytic rates and the allocation of enzyme abundances across reactions [2,3].

After the change:

While already useful for predicting a wide range of phenotypes, the predictive performance of GEMs has been further improved by integrating protein constraints, such as for enzyme catalytic rates and the allocation of enzyme abundances across reactions [2,3]. Enzyme abundances are central to metabolic function, since they impact the rate of reactions and regulation of pathways, with implications to biotechnology and medicine [4].

Before the change – L16-24:

Protein allocation determines the activity of cellular pathways and affects growth across all organisms. Therefore, different experimental and machine learning approaches have been developed to quantify and predict protein abundances, respectively. Yet, despite advances in protein quantification, it remains challenging to predict condition-specific allocation of enzymes in metabolic networks. Here we propose a family of constrained-based approaches, termed PARROT, to predict how much of each enzyme is used based on the principle of minimizing the difference between a reference and an alternative growth condition using protein-constrained metabolic models.

After the change:

Protein allocation determines the activity of cellular pathways and affects growth across all organisms. Therefore, different experimental and machine learning approaches have been developed to quantify and predict protein abundance and how they are allocated to different cellular functions, respectively. Yet, despite advances in protein quantification, it remains challenging to predict condition-specific allocation of enzymes in metabolic networks. Here, using protein-constrained metabolic models, we propose a family of constrained-based approaches, termed PARROT, to predict how much of each enzyme is used based on the principle of minimizing the difference between a reference and an alternative growth condition.

2. Line 101-102: "...the optimal growth state is disturbed". The authors need to define/describe what they refer to as the optimal growth state and the associated disturbance to it.

Thank for pointing this out. We intended to state that the optimal growth state is the growth condition where the cells grow with optimal conditions, such as optimal temperature, optimal pH, optimal nutrient availability, optimal aeration, optimal agitation, and so forth. The associated disturbance can be understood to be any deviation from this. We rephrased this section to refer to an initial growth condition to avoid confusion.

Before the change – L100-104:

Moreover, as cells are exposed to stresses or changing environmental conditions, the optimal growth state is disturbed, leading to an alternative growth state in which gene expression, regulatory pathways and metabolic flux are changed in adjusting the cell to this new physiological condition [16].

After the change:

Moreover, as cells are exposed to stresses or changing environmental conditions, the current growth state is disturbed, leading to an alternative growth state in which gene expression, regulatory pathways and metabolic flux are changed in adjusting the cell to the new physiological condition [16].

3. In the results section, the authors provide a count range of the enzymes by each model and indicate that since this count is similar to the experimental count, their model is performing better than the counterpart (pFBA). However, it is unclear how are count ranges alone sufficient to ascertain a better performance. Shouldn't the actual enzymes, themselves, be matching or similar? Alongside, the associated protein allocation counts also need to be accounted for, isn't it?

Thanks for mentioning this. Actually, we make no claims of model performance based on the count of enzymes themselves. We stress that we do use the associated enzyme allocation values for the comparisons and for the correlations we calculate, which is how we derive all conclusions of this work. We merely state the count of enzymes so the reader can have a better understanding of how the correlations were obtained and how reliable they are, since the sample size affects correlations.

4. In lines 177--180, the authors mention their reasoning about their choice of the dilution rate while accounting for aerobic growth and metabolic shift prevention. This also raises a concern about the operational ranges for each of the 4 prediction models. The authors should describe the conditions that need to be satisfied for their proposed model/s to be suitable for these predictions.

Thanks for raising this concern. We state that we have chosen the smallest dilution rate to account for aerobic growth and to prevent metabolic shifts because we were comparing different timepoints of a chemostat experiment with increasing growth rates. The smallest dilution rate represents the steady-state condition with the most stable phenotype to perform these comparisons. However, this does not mean that this is a limitation of the operational ranges of PARROT, nor it is limited by any other phenotype. Additionally, we used data from many growth conditions obtained from batch cultivations, where these concerns do not apply. We stress that PARROT can be used between any pair of growth conditions, on the assumption that one condition is the initial steady state condition with a stable phenotype and/or optimal growth (optimal temperature, optimal pH, optimal nutrient availability, optimal aeration, optimal agitation, and so forth), and the other condition is the steady state condition obtained after the initial condition is changed, reflecting an adaptation of enzyme usage and flux distribution in this new condition.
